# Making sense of joint commissioning: three discourses of prevention, empowerment and efficiency

**DOI:** 10.1186/1472-6963-13-S1-S6

**Published:** 2013-05-24

**Authors:** Helen Dickinson, Jon Glasby, Alyson Nicholds, Helen Sullivan

**Affiliations:** 1 Health Services Management Centre, University of Birmingham, UK; 2 Middlesex University Business School, London, UK; 3 School of Social and Political Science, University of Melbourne, Australia

## Abstract

**Background:**

In recent years joint commissioning has assumed an important place in the policy and practice of English health and social care. Yet, despite much being claimed for this way of working there is a lack of evidence to demonstrate the outcomes of joint commissioning. This paper examines the types of impacts that have been claimed for joint commissioning within the literature.

**Method:**

The paper reviews the extant literature concerning joint commissioning employing an interpretive schema to examine the different meanings afforded to this concept. The paper reviews over 100 documents that discuss joint commissioning, adopting an interpretive approach which sought to identify a series of discourses, each of which view the processes and outcomes of joint commissioning differently.

**Results:**

This paper finds that although much has been written about joint commissioning there is little evidence to link it to changes in outcomes. Much of the evidence base focuses on the processes of joint commissioning and few studies have systematically studied the outcomes of this way of working. Further, there does not appear to be one single definition of joint commissioning and it is used in a variety of different ways across health and social care. The paper identifies three dominant discourses of joint commissioning – prevention, empowerment and efficiency. Each of these offers a different way of seeing joint commissioning and suggests that it should achieve different aims.

**Conclusions:**

There is a lack of clarity not only in terms of what joint commissioning has been demonstrated to achieve but even in terms of what it *should* achieve. Joint commissioning is far from a clear concept with a number of different potential meanings. Although this ambiguity can be helpful in some ways in the sense that it can bring together disparate groups, for example, if joint commissioning is to be delivered at a local level then more specificity may be required in terms of what they are being asked to deliver.

## Introduction

As many of the papers in this supplement illustrate, commissioning has become a key concern of the English national government in recent years. This concept is at the heart of the government’s current health reforms and has also been employed across an array of other policy domains see [1]. The issue of how different government agencies work together in a more coordinated way is not a new one, but since the late 1990s has gained increased impetus becoming a central feature of the New Labour government’s policy [2]. Combining the two agendas of commissioning and joint working, policy has increasingly started to focus on the need for greater joint commissioning of health and social care [see, for example, [3,4]. Broadly speaking joint commissioning is concerned with the ways in which relevant organizations might work together and with their communities to make the best use of limited resources in the design and delivery of services and improve outcomes. Yet, the current policy rhetoric about the importance of joint commissioning often seems to lag behind the reality at ground level - despite the fact that aspirations for effective joint commissioning date back many years see, for example, [5]. At least part of the difficulty seems to lie in the fact that joint commissioning is, by definition, more complex than commissioning in single agency settings; joint commissioning almost inevitably brings additional challenges because of the need to develop effective partnerships between health, social care and beyond.

The New Labour governments of 1997-2010 were clear that greater inter-agency collaboration was necessary to provide seamless services for users and carers see, for example,[6,7] , and made a commitment to achieving ‘joined-up solutions' to ‘joined-up problems' [8]. Despite the focus on greater competition in some areas of the health service, this emphasis on the importance of collaboration (or ‘integrated care’) has continued under the current Coalition government [4,9,10] , with an added need to respond to a difficult financial context by working together more effectively and using scarce resources to best effect. Responding to these policy developments, a large number of different partnership arrangements have been developed in different parts of the country (e.g. Care Trusts, joint appointments, the use of staff secondments and joint management arrangements and Joint Strategic Needs Assessments). Although there is a substantial and growing literature on partnership working see, for example, [2,11-13] , there are a number of limitations to our existing knowledge. Chief amongst these is that much of the current literature is descriptive and ‘faith-based', emphasising the virtues of partnership working without necessarily citing any evidence for the claims made see, for example, [14-16] . Often, the focus is on the *processes* of partnership working (how well do we work together), rather than whether or not this improves *outcomes* for people using services. One of the difficulties encountered in examining outcomes is that it is not always clear just what types of outcomes joint commissioning should be delivering. Although health and social care policy contains a number of calls for more and better joint commissioning, beyond some rather abstracted notions of creating “better” services and outcomes for service users there is little specificity about just what this would look like in practice.

This paper reviews the literature in order to examine the types of claims that have been made for joint commissioning in terms of what it should achieve in practice. There is a distinct lack of high quality empirical evidence about joint commissioning and so we adopt an interpretive approach which identifies a series of discourses from the literature, each of which suggests that joint commissioning should achieve different things. The paper starts by explaining what is involved in an interpretive review in more detail before moving on to set out the findings of the review. We identify three different meanings that joint commissioning seems to have within the literature; prevention, empowerment and efficiency. The final discourse of efficiency is the one probably most closely associated with the market reform agenda which is at the centre of this supplement. We conclude that ultimately this discourse may be limited in practice by the agency of the individuals who are involved in implementing joint commissioning at the local level.

## Interpretive analysis

The focus of mainstream policy analysis has arguably tended to be on generating rigorous quantitative data, objectively separating facts and values and searching for generalisable findings which have validity outside of the social context they were forged in [17]. In this sense, policy analysis has often been seen as a ‘rational model’ that might inform decision-making - or as Stone [18] terms this, the “rationality project”. Postpositivist approaches reject the notion of “traditional scientific principles” and the idea that a unified understanding of scientific methodology can be applied to all research questions [19]. Everyday life is understood as embedded in social and cultural meaning which is produced (and reproduced) by discursive practices which are outside of actors’ choosing or making [20]. An interpretive approach recognises that the social world is not fixed and objective but is framed through discourses of actors. Interpretive approaches argue that it is important that we consider socio-cultural processes with the analysis of policy and the way that individuals make sense of their every day lived experience. Fischer [20][ p. 49] argues that ‘rather than seeking proofs through formal logic and empirical examination, the investigation of social action requires the use of metaphoric processes that pull together and connect different experiences based on perceived similarities’. Through our review of the literature we sought to identify a series of discourses which frame joint commissioning in slightly different ways in terms of the problem that joint commissioning is attempting to achieve, the types of activities that it seeks to do this through and the impacts that this should have in practice.

Discourses are essentially a system of meaning, “an ensemble of ideas, concepts and categorisations through which meaning is allocated to social and physical phenomenon, and which is produced and reproduces in an identifiable set of practices” [21][ p. 45] . Discourses comprise “all practice and meanings shaped by a community of social actors” [22][p. 5] and “are revealed as narratives, rhetorical strategies, organisational metaphors, traditions, collections of storylines, and cognitive normative frames” [23][pp. 46-47]. Over time discourse may become ‘sedimented’ meaning that they are ‘taken for granted’ and influence the ways in which actors perceive the conditions of possibility and their consequent room for manoeuvre [24]. In this way discourses that are seemingly neutral, for example scientific discourses concerning the causes of poverty, may in fact actually fix particular subjectivities [25]. In this way maximum social control may be gained with a minimum expenditure of force and Foucault e.g. [26,27] provides examples of this through schools, factories, asylums, military barracks and others.

As will be described in further detail below, the extant literature is far from clear cut about the impact of joint commissioning but also in terms of what its purpose is. As such, this research focused on the different sorts of meanings that are given to joint commissioning within the literature. Discussions of issues or evidence relating to joint commissioning rarely appeared in the context of the language of discourse, but instead these are our interpretations on the basis of the sorts of language used, the drivers, motivations and contexts for joint commissioning used in articles. In the following section we say more about how these discourses were extrapolated in the context of the wider search strategy.

## Search strategy

This review formed the scoping stage of a wider project investigating the definitions, processes, services and outcomes of joint commissioning [28]. The purpose of the literature search was to examine the extant literature and identify evidence of the kinds of outcomes of joint commissioning that have been observed. The literature search also sought to identify examples of best practice in regards to joint commissioning which might then be examined in the case study component of the research see, [28]. The team searched a number of databases covering health and social care including: HMIC; Medline; ASSIA; Pro-quest/ EBSCO; Social Care Online; Social Sciences Citation Index; Social Services Abstracts; and ISI Citation Index database. The search terms used for this exercise were (partnership* OR joint working OR integrated working OR inter-agency working) OR (commissioning OR joint commissioning) AND (good practice OR best practice OR innovation OR success). There were no date restrictions applied but papers needed to be written in English to be included.

In total this search retrieved 512 abstracts which were read and the inclusion and exclusion criteria applied. Articles were included where they explored joint commissioning in its broadest sense (i.e. more than one organisation involved in needs analysis and subsequent purchasing of services) and based on an English context. Following this process, 399 items were rejected due to a lack of relevance. The majority of the items that were rejected mentioned joint commissioning in passing, but this was not the central concern of the article or an issue that was addressed in any real detail. Seven of the items sought were not able to be obtained, others proved irrelevant once the full item was obtained and in the process of reading full items others were collected through ‘snowballing’ sources (see Table 1). A final list of 105 items was constructed and these items were retrieved in full. Two independent members of the research team read 10 items selected at random and used a standardised pro forma to extract relevant data from these articles. These pro formas were compared for their inter-researcher reliability and then the remainder of data extraction completed.

**Table 1:** Numbers of items retrieved in literature search

Stages of literature review process	Number
Abstracts identified from initial search of database	512
Abstracts discarded after application of inclusion and exclusion criteria	399
Duplicates	4
Items unable to obtain	7
Items discarded after reading in full due to lack of relevance	4
Additional items found through snowballing	7
**Total number of items included in review**	**105**

Many of the items identified through this search process derive not from the peer review literature, but instead from practice and policy literatures and this has implications in terms of the methodologies adopted in these pieces and the status of this evidence and this is considered in some detail in the following section. To conclude this section we provide detail of how we extrapolated the different discourses through the process of analysis.

The approach adopted in this research follows the previous work of Dickinson [29], Williams and Sullivan [30] and Sullivan et al [24], which all adopt an interpretive approach to understanding the concept of collaboration more generally. The standardised proforma that was used in the literature review included sections on the aims and aspirations of joint commissioning activities, what activities are involved in joint commissioning and how joint commissioning would be delivered. Not every item provided detail on all of these issues, but where this was included this was noted in the proforma. In undertaking this review we drew on the work of Yanow e.g. [31,32] who suggests that in identifying discourses it is important to pay attention to language in terms of the symbols and metaphors alluded to and the sorts of objects and acts which a particular policy is to be implemented by. Once all the items had been coded in this way we drew together the themes in order to identify the different “interpretive communities” [33] p.20. In keeping with the goals of an interpretive approach to surface implicit meaning, it is possible to see how despite a common reference that joint commissioning should lead to ‘service improvement’ in a general sense, there were differences in the language, objects and acts used to describe how joint commissioning is actually done. Through subsequent consolidation of the various activities and themes, we identified three different ways that the literature frames joint commissioning, each of which is constituted by different uses of language, processes and practices used to implement and communicate policy. We say more about these discourses in detail below, first we reflect on the general nature of the evidence base generated by the review.

## The nature of the evidence base

Although attempts were made to uncover as many papers relating to joint commissioning as possible, it quickly became apparent that the joint commissioning literature is not terribly robust in the sense that there is not a good body of peer review literature underpinning this concept. Of the total papers selected, only a small number were peer-reviewed articles (27). Of the remainder, 42 were practice-based review articles, most of which formed the basis of commentaries and reflections on joint commissioning, eight were publications from think tanks and 26 papers were government documents describing policy initiatives (see Table 2). Thus, despite joint commissioning having been a key component of health and social care policy for some time, there appears to be little good quality (i.e. peer reviewed) evidence relating to this concept. What evidence that does exist is predominantly in the form of governmental publications which often lacked a clear evidence base or a systematic approach to generating evidence about joint commissioning.

**Table 2:** Types of items retrieved in literature search

Type of article	Total number found	Percentage of total items retrieved
**Practice-based journals**	42	40
**Peer reviewed journals**	27	26
**Government documents**	26	25
**Think Tank and independent policy advice**	8	8
**Book chapters**	2	2
**TOTAL ITEMS RETRIEVED**	105	

Our first conclusion about the extant joint commissioning literature is that there is a distinct lack of high quality research evidence, with much of the literature comprising opinion pieces or the voices of those who have been involved in leading these types of initiatives. To some extent we might expect this given that similar claims have been made about the commissioning literature more generally. For example, in a review of the evidence base of published generic social care commissioning guides Huxley and colleagues [34] found that although these guides were accessible in terms of being clear and well written, the evidence was drawn mostly from government documents rather than research evidence. Similarly, Dickinson [16] and Bovaird *et al *[1] also observe that there is a lack of robust evidence pertaining to collaboration in health and social care and commissioning more generally.

On closer inspection of those articles that appear in peer reviewed journals (Table 3), not only do we see that the methods are largely qualitative (33%), the large majority comprise a case study approach (41%) which provide practical examples of joint commissioning in a particular situation. Often these were very descriptive accounts of activities at one site without theorisation or an attempt to extrapolate to a wider context. Where case study methods were used, there is rarely, if any, discussion about the methods used to gather data, or how the sample was drawn. Of the three studies reported which adopt a ‘mixed methods approach’ (11%) these are actually linked publications which all draw on the same bank of data involving quantitative survey and qualitative interviews to offer different perspectives of the process of joint commissioning. The remainder of the literature constitutes literature reviews and editorials which provide rigorous accounts of the data which already exists around partnership, but which lack any empirical contribution of their own.

**Table 3:** Methods used in peer reviewed literature on joint commissioning

Methodology	Number	References
Mixed methods (qualitative & quantitative)	3	[61-63]
Qualitative	9	[37,64-71]
Quantitative	0	N/A
Literature reviews	2	[15,72]
Case study	11	[53,73-82]
Other	2	[83,84]
**TOTALS**	**27**	

On reading the joint commissioning literature the first thing that becomes immediately apparent is that definitions of this term are rather sparse. Joint commissioning is frequently referred to as though we all know what this term means and there is little need to define this in any further detail. On further investigation we see that joint commissioning is often conflated with other forms of joint working such as; partnership, integration or collaboration [35-38]. Joint commissioning is often discussed in a similar way to these other terms with no clarification about if and how this differs to other forms of joint working. Broadly speaking joint commissioning is concerned with the ways in which relevant organizations might work together and with their communities to make the best use of limited resources in the design and delivery of services and improve outcomes. However, given that this is potentially a wide aim, there is a lot of elasticity in terms of what joint commissioning actually is and debates surrounding the meaning, purpose and impact of joint commissioning make for an increasingly complex and confusing debate [38,39] . In attempting to define this concept Rummery and Glendinning [40] state that “there is no universally agreed definition of joint commissioning; the term can cover a wide range of activities” (pg. 18). Williams and Sullivan [30] go further than this arguing that joint commissioning does not actually have a single meaning, but that several communities of meaning co-exist and each aims to deliver different types of outcomes.

Given that joint commissioning is such a “broad and malleable concept that it can legitimately mean different things to different people” [41] cited in [42] p. 18 , rather than attempting to interrogate a rather limited literature which does not deal in any systematic way with the issue of outcomes, as explained above, we adopt an interpretive approach to investigate the various claims that are made for joint commissioning, based on different understandings about what it means and what it should achieve in practice. In doing so we move away from treating joint commissioning as an overly-rational tool of policy. What this means is that instead of treating joint commissioning as a simple means-ends tool, we seek to explore the additional work that this concept might do beyond those which are articulated by their makers (i.e. policy makers and senior leaders of health and social care agencies). In employing this process of analysis it is possible to surface three different ways in which joint commissioning is discussed in the literature: prevention, empowerment and efficiency (summarised in Table 4). It is to these discourses that we now turn.

**Table 4:** Discourses of joint commissioning identified through literature search

	Joint commissioning as prevention	Joint commissioning as empowerment	Joint commissioning as efficiency
**What joint commissioning should achieve**	Deliver preventative services through early intervention. This should in turn reduce inequalities, improve the quality of services and make services more accessible.	This should involve patients, service users and carers in the co-production of services. A user-led approach to care should be adopted that promotes self-care and in doing so transforms health and social care away from being professionally-led.	What is important is improving efficiency and reducing waste and duplication in health and social care services. In turn this should also improve access and performance of services.

**Organisational processes to promote joint commissioning**	Service re-design is important here and thinking about the needs of individuals and providing services around these. A key role for the alignment of strategies and budgets and the development of care pathways.	Personalisation of services plays an important role here with service users being given budgets with which to determine their own care. Fairness, inclusion and respect should be at the heart of all processes.	Increasing the number of providers that are available to health and social care commissioners will give more choice and competition. Greater freedoms and flexibilities for providers and the freedom to innovate should be supported by incentive-based reward and quality will be assured through inspection.

**Organisational practices that support joint commissioning**	The focus here is around commissioning practices and making full use of the Joint Strategic Needs Assessment to identify gaps in need.	What is important is how we work with service users and carers and the management of complex relationships. Workforce development and training may help with this.	More effective management of information may help to identify waste. What is important is the relationship with providers of care and how these are contracted with and performance managed.

## Joint commissioning as prevention

The first discourse refers to joint commissioning in the context of prevention and early intervention. The purpose of joint commissioning then is around health improvement through the reduction of inequalities. Common to this way of seeing is a focus on improving the ‘quality’ of service provision as a basis for improving the health and well-being of populations e.g. [3,43,44] . The view that joint commissioning offers a means of prevention and early intervention is evident in policy documents which talk about organisations needing to work together to promote health and reduce inequalities by improving the quality and accessibility of services [3,43,45] . For instance, in a service review of the joint commissioning of drug treatment services which noted a wide variation in quality, the former National Drug Treatment Agency talks about the need to “improve the health and well-being of service users and their families and...reduc[e] crime related to their substance misuse” [44] p. 3 . Similarly, in the *Commissioning framework for health and well-being* the focus is on “involving the community to provide services that meet their needs, beyond just treating them when they are ill but also keeping them healthy and independent” [3] p.7.

The use of the joint strategic needs assessment for the local area is crucial in informing where the gaps are in terms of local provision and needs. The effective design of care pathways can be helpful in aligning needs of the local population with the provision of services. Policy programmes which have the prevention of ill-health and early intervention at their heart tend to allude to the notion of ‘service re-design’ as a means of achieving policy goals. This is premised on a belief that inequalities in service provision can be addressed by finding ways of improving how services are delivered [3,45]. In noting the tendency for “a small number of people to incur extremely high cost”’ in health and social care provision, the Birmingham Total Place Pilot shows how the total public expenditure could be re-aligned by moving away from separate strategies and financial plans towards financial planning with longer term investment in mind [46] p.7. Similarly, in the context of children’s services, there is an emphasis on commissioners working together to commission children’s services to “ensure that children and young people’s services meet population requirements and address health inequalities” [47] p. 12.

The focus on prevention and early intervention is driven by efforts to identify gaps in service provision through the better management of commissioning practices such as joint strategic needs assessment and the development of care pathways. The sorts of practices that are referred to in this discourse are interventions such as health needs assessment and performance management devices (i.e. self assessment toolkits). For instance, the National Treatment Agency for Substance Misuse, suggests that energy is invested *through improving the efforts of local drug partnerships* to improve their “commissioning management”; (that is how services are planned, procured and managed) and harm reduction serviced (which reduce the risk of blood borne viruses) *and through health needs assessment* and the development of indicators to assess the effectiveness of commissioning. What is important in the context of this discourse is that commissioners better understand the complexities of care pathways for their client group and ensure they are better able to pin-point where there might be gaps so that resources can be focused on the needs of client groups.

## Joint commissioning as empowerment

In contrast to the first discourse with its focus on organisation-led service change, the second sees the purpose of joint commissioning in the context of user-led service change based around the promotion of self care [48-50]. Here, language tends to focus on meeting the needs of service users and carers through the co-production of their own care and the empowerment that this is believed to bring. This involves a fundamental shift in power relationships from paternalistic services to ones truly driven by service users. Here, the language in policy documents appears to centre on adopting a more ‘user-led’ approach to joint commissioning based around involving patients, service users and carers’ in the co-production of services [48,49] . For instance, in the *Working Together for Change* policy [44] this is expressed in terms of transforming adult social care away from professionally-led service delivery towards a user-led model which involves the design, commissioning and evaluation of individual services. Such an approach is bound up in the core values of the personalisation agenda (see Catherine Needham’s paper in this supplement for more information on this [51]) where “the services people use are based on their circumstances, need, preference and desired outcomes” [52] p. 5. The idea here is that “if service users are able to direct their support in a truly personalised way then joint commissioning is needed to effectively manage markets and provide the support that these micro commissioners need”.

In reviewing the key activities in commissioning social care, the Care Services Improvement Partnership defines effective commissioning as “care that adds maximum value for patients in a system that promotes fairness, inclusion and respect from all sections of society” [53] p. 11 . This is also picked up in some of the earlier practice-based literature which notes how the advent of partnerships brought with it a focus on the inclusion of service users, carers and a much wider range of agency partners [54]. This shift in focus towards service users/ carers also highlights the importance of the commissioning cycle as a policy tool in managing a mixed economy of care. Here, government documents highlight the importance of the commissioning cycle in responding flexibly to changes in the “demographic and epidemiological” service needs of a population over time [55] p. 2. From the perspective of an empowerment approach then, an important policy object involves the commissioning cycle in ‘driving service change’ and ensuring that care packages are responsive to need [49]. This is in stark contrast to the preventative discourse which relies on health needs assessment as a means of assessing individual needs, resources, markets and services available.

In terms of the practices associated with this discourse there is a noticeable shift in emphasis towards the importance of partnerships in managing the “complex inter-relationships between the varying roles, responsibilities, resources and traditions of the many agencies involved in delivering health and social care services” [54] p. 193. Gostick also talks about the value of partnerships in “managing a mixed economy of care” [54] p. 196. But the emphasis here is on partnerships between agencies, service users and their carers “so that services fit around the service user and the transformation that needs to take place”. This is most keenly expressed in the *Working to Put People First* strategy [48] p.1 which talks about “putting choice and control into the hands of people who use adult social care”. This focus on partnership with service users implies a need to develop the capacity of the workforce in ensuring that staff have the right skills to work in a collaborative way. Hence, the focus of the *Working Together for Change *[49] policy highlights the need for effective leadership and workforce development in supporting staff from a range of backgrounds to adapt to a more complex and personalised joint commissioning environment.

## Joint commissioning as efficiency

This third discourse frames joint commissioning in the context of efficiency. Here, language is rooted in a concern to meet rising expectations from the public about what services they should receive and the quality of these. A key concern here is how we can improve access to health and social care services by increasing choice and control. There is a tendency here to focus on increasing the range of alternative providers to give service users choice and drive competition. Concern about reduced waiting times and high quality care centred on patients is premised on beliefs that in the past, hospitals and providers have taken for granted the funding they have received, irrespective of clinical performance or the quality of outcomes produced [56] . Implicit within this way of seeing, is the need to provide patients and people with more choice and control over their health and care and clinical staff with the means to meet these rising expectations. For staff this is expressed in the notion of greater freedoms and flexibilities in an attempt to improve “clinical services and productivity” [56] p.6. Interestingly, such notions of ‘efficiency’ also express the full weight of the marketisation of health and social care provision by promoting the concept of ‘choice’, not in terms of patient choice, but in the context of seeking a ‘wider range of providers’ and their measurable performance in delivering outcomes. This is in direct contrast to the discourse of prevention (where choice refers to patient choice about service delivery) or empowerment (in ensuring services are more user-led). Here, increasing patient ‘choice’ appears to mean ‘opening up the market’ to a greater range of alternative providers.

The means of achieving such increased choice is perhaps most keenly reflected in the government document *Creating a patient-led NHS: Delivering the NHS Improvement Plan *[57]. Here the means of improving such ‘access’ lies with a complete overhaul of the way in which health and social care services are expressed at the point of delivery. Such reform is a central component of the *NHS Plan *[58] and *NHS Reform *[56], which specifically, proposed four work-streams to implement demand side reforms; supply side reforms, transactional reforms and systems management. These four ‘streams’ of improvement are described as:

*“more choice and a much stronger voice for patients* (*demand-side reforms*)*; more diverse providers*, *with more freedom to innovate and improve services* (*supply-side reforms*)*; money following the patients*, *rewarding the best and most efficient*, *giving others the incentive to improve* (*transactional reforms*)*; system management and decision making to support quality*, *safety*, *fairness*, *equity and value for money* (*system management reforms*)*” *[56] p. 5.

This linking of payments to patient experience and health outcomes is part of the drive to incentivise better clinical performance. Better management of information is also seen as key in ensuring that the perceived goals of joint commissioning (in terms of integrating services, reducing duplication and waste and reducing waiting times) are all met in a satisfactory way [59,60] .

## Concluding discussion

Joint commissioning is a key element in the English Government’s reform of public services, including but going far beyond, health and social care services. However, health and social care services remain a particularly interesting case to explore because attempts at improving co-ordination between these service areas have a long history and joint commissioning was introduced into health and social care before any other policy areas. At the same time, as we have illustrated in this article, clarity about what joint commissioning is remains elusive in the literature and evidence of its impact on improving service co-ordination and outcomes is at best partial. On the basis that developing an improved understanding of the meaning(s) of joint commissioning might help facilitate greater understanding of how to look for any impact on outcomes; we undertook an interpretive review of the available literature. This generated three distinct discourses of joint commissioning amongst policy makers as: prevention, empowerment and efficiency. This finding is in line with those of Williams and Sullivan [61] who suggested that interpretive approaches can reveal the coexistence of different communities of meanings, associated with the interaction of multiple actors and interests to achieve a plurality of outcomes.

However we think that our analysis can be taken further to reveal more than simply the co-existence of different discourses of joint commissioning amongst policy makers. Examining the content of each of the discourses separately and together reveals the way in which they offer a means of de-politicising decisions about health and social care resources and services. De-politicisation is itself a political act which aims to redefine decisions that should properly be the subject of democratic debate and discussion as matters of management and process. In so doing it also disguises or excludes key considerations from these reconstituted management decisions.

The discourse of prevention is framed in the context of promoting health and reducing inequalities. It suggests that the development and employment of specific technical or management processes, such as joint health needs assessments or joint performance management regimes, can improve our understanding of what local communities need. More importantly, it can enable professionals to offer more co-ordinated and coherent care that is of better quality and hence will have a bigger impact on preventing poor health and limiting social care needs in the future amongst deprived or marginalised communities. Throughout, this discourse emphasises the use of management instruments and processes. What it fails to allow for though is the way in which poor health and inequalities are shaped by wider socio-economic conditions, conditions that are beyond the reach of performance planning and management regimes with their focus on improvement through use of existing resources. The implicit message here is that inequalities can be addressed simply by virtue of better use of resources facilitated by joint commissioning. There is no acknowledgement that a deeper set of structural conditions may require additional resources to be invested over a long period of time to deal with prevailing conditions.

The empowerment discourse offers an alternative vision, one in which joint commissioning is a means of securing user involvement or control through personalisation. The consequence of such involvement or control, are more responsive and appropriate services for individual users. The emphasis here is on joint commissioning as a means of promoting user voice and choice. However, here too, there are important considerations which are absent. The persistent focus on improving individual care and experience of service offers the implicit and sometimes explicit hope that through joint commissioning all needs will be met. Matters of rationing and limited resources are not considered in the communities of meaning and there is no acknowledgement that a reformulation of health and social care budgets along these lines will have negative consequences for some services users.

Finally the discourse of efficiency focuses on joint commissioning as a way of reducing bureaucracy and waste. Arguably it is this discourse which is closest to the kinds of ‘market-based reforms’ that are the focus of this supplement. The efficiency discourse suggests that working together to plan, design and deliver services will enable health and social care professionals to identify and remove duplication and blockages. This is deemed to happen through a combination of better management processes but importantly here the emphasis is on the exercise of professional judgement as a route to greater efficiency. What is not addressed in this discourse is the inevitable persistence of some ‘waste’ and bureaucracy in any system of decision making, sometimes for good reasons, e.g. the need to build in redundancy, or the need to have an alternative perspective.

Separately and together these discourses of joint commissioning provide a means of managing the demand side of welfare that is absent from political decision making and dispute. Aside from the obvious concern that this may raise about the limited and limiting role afforded to democratic politics, there are other reasons why the use of joint commissioning as a means of depoliticising decisions in health and social care might be undesirable. Central among these is the fact that to date there is relatively little evidence that joint commissioning does deliver improved services and outcomes and that this is situated amidst a lack of evidence in terms of health and social care and commissioning more generally [1,16]. In addition the use of an interpretive framework to explore what and how joint commissioning means amongst policy makers provides a further reason to be cautious.

The analysis has identified three distinct discourses which we have suggested co-exist in the literature. However they may not comfortably co-exist in practice. For example, there are likely to be tensions between the user focus of the empowerment discourse and the technical orientation of the prevention discourse or the privileging of the professional in the efficiency discourse. In practice discourses are likely to conflict and local conditions will result in one prevailing over others. Local conditions offer a very important counter to the direction of the discussion up to this point. As we have indicated, our analysis has concentrated on the discourses in operation amongst the policy elite. Not accounted for in this analysis so far is what happens to joint commissioning and how it is interpreted when local professionals, managers, practitioners and service users are engaged. As agents with differing resources at their disposal they are likely to try and make sense of joint commissioning in ways that are particular to and in keeping with their own values. This not only opens up the possibility of new discourses being generated, but it also allows for the expression of resistance to the discourses of the policy elite. One consequence of this may be deliberate attempts to re-politicise both the policy and practice of joint commissioning. As such the discourse of efficiency which gives voice to market based reform in the context of the joint commissioning literature may be limited in terms of its interpretation by local areas.

## Competing interests

None.

## Authors’ contributions

HD led on the writing of the paper and the literature review. JG was project PI and assisted with editing paper. AN assisted on reviewing of literature and analysis and writing up of discourses. HS contributed in terms of methodology and analysis of discourses and involved in writing of paper.

## Declarations

Publication of this supplement was funded by the Nuffield Trust.

This article has been published as part of *BMC Health Services Research* Volume 13 Supplement 1, 2013: The Limits of Market-based Reforms. The full contents of the supplement are available online at http://www.biomedcentral.com/bmchealthservres/supplements/13/S1.
